# The Role of Metabolic Enzymes in the Regulation of Inflammation

**DOI:** 10.3390/metabo10110426

**Published:** 2020-10-26

**Authors:** Wesley H. Godfrey, Michael D. Kornberg

**Affiliations:** Department of Neurology, Johns Hopkins University School of Medicine, Baltimore, MD 21205, USA; wgodfre1@jhmi.edu

**Keywords:** immunometabolism, inflammation, metabolism

## Abstract

Immune cells undergo dramatic metabolic reprogramming in response to external stimuli. These metabolic pathways, long considered as simple housekeeping functions, are increasingly understood to critically regulate the immune response, determining the activation, differentiation, and downstream effector functions of both lymphoid and myeloid cells. Within the complex metabolic networks associated with immune activation, several enzymes play key roles in regulating inflammation and represent potential therapeutic targets in human disease. In some cases, these enzymes control flux through pathways required to meet specific energetic or metabolic demands of the immune response. In other cases, key enzymes control the concentrations of immunoactive metabolites with direct roles in signaling. Finally, and perhaps most interestingly, several metabolic enzymes have evolved moonlighting functions, with roles in the immune response that are entirely independent of their conventional enzyme activities. Here, we review key metabolic enzymes that critically regulate inflammation, highlighting mechanistic insights and opportunities for clinical intervention.

## 1. Introduction

Immunologic responses are complex and finely tuned. Immune cells of multiple types must integrate an array of external signals and coordinate with one another to produce responses that are appropriately targeted and effective, leading to profound yet precisely timed changes in proliferation and function. The past two decades have produced recognition that cellular metabolism, previously relegated to a housekeeping role, in fact critically regulates immune functions. Immunologic signals produce a broad reprogramming of metabolic pathways, which drives changes in immune cell activation, differentiation, and effector functions. This regulation of immune responses by metabolic pathways, termed “immunometabolism”, has become a major focus of research, with a goal of identifying pathways that can be targeted in human diseases, such as autoimmune diseases characterized by dysregulated inflammation.

Our mechanistic understanding of the role of metabolism in immunology is ever growing, but certain common principles have come into focus. Metabolic reprogramming appears necessary to provide precursors and meet the energy demands unique to specific immunologic states. Moreover, metabolites themselves can act as signaling molecules that directly modulate inflammatory responses. Within this framework, metabolic enzymes have been identified that regulate inflammation by controlling flux into key pathways and/or the altering levels of immunoactive metabolites. As an example of the beauty and efficiency of evolution, several such enzymes have been co-opted as “moonlighting” proteins, playing roles in immunologic signaling pathways entirely independent of their conventional enzyme activities. As critical regulators of inflammation, these enzymes represent potential therapeutic targets in human disease. Animal and, in some cases, human studies support the plausibility of pharmacologically targeting metabolic enzymes to modulate immunity [1].

Here, we will review the growing list of metabolic enzymes that critically regulate immune responses, highlighting their susceptibility to pharmacologic interventions in animals and humans. Given the breadth of metabolic processes shown to impact immune function, we have structured our review based on several key metabolic pathways.

## 2. mTOR and AMPK—The Master Regulators of Metabolism

Mechanistic target of rapamycin (mTOR) is a serine/threonine kinase with major regulatory powers over cell survival, growth, and metabolism. mTOR, which can exist within two distinct complexes (mTORC1 and mTORC2), integrates information about cellular energy and nutrient availability with external stimuli to produce broad effects, including the induction of nutrient transporter expression, promoting the activity of glycolytic enzymes, increasing lipid synthesis, inducing ribosome synthesis, and favoring the transcription and translation of various proteins [2]. Indeed, the mechanisms by which mTOR induces metabolic reprogramming both within immune cells and beyond are innumerable, and a full accounting is beyond the scope of this review. However, it is crucial to note that mTOR acts as a master regulator of immune responses, positioning both lymphoid and myeloid cells for the pro-inflammatory state by inducing broad metabolic changes. In T cells, mTOR activation occurs downstream of AKT/PI3K signaling in response to the co-stimulation of the T cell receptor (TCR) and CD28 [3]. In myeloid cells, mTOR is activated downstream of Toll-like receptors (TLRs) and cytokine receptors [4]. Within both myeloid and lymphoid cells, mTOR signaling is critical for the upregulation of metabolic pathways required for inflammatory activation and effector functions, including glycolysis, the pentose phosphate pathway, and glutaminolysis, acting largely through the transcription factors HIF-1α and Myc. Rapamycin, a well-known inhibitor of mTOR, is used as an immunosuppressive drug to prevent organ transplant rejection [5].

Signaling downstream of mTORC1 and mTORC2 differentially regulates CD4^+^ T cell differentiation. A deficiency of mTOR, which impacts both complexes, impairs the differentiation of T helper (Th) 1, Th2, and Th17 cells, while promoting the differentiation of regulatory T (Treg) cells [6]. Both mTORC1 and mTORC2 inhibit Treg differentiation, and a deficiency of both complexes is required for enhanced Foxp3^+^ Treg production. The selective impairment of mTORC1 signaling through a deficiency of its upstream regulator Rheb was found to prevent Th1 and Th17 differentiation while promoting Th2 differentiation, while a reciprocal effect on differentiation was observed with mTORC2 deficiency [7]. However, another study found that CD4^+^ cells deficient in mTORC2 failed to differentiate into either Th1 or Th2 cells [8].

The counterbalance to mTOR in metabolic regulation is AMP-activated protein kinase (AMPK), which reciprocally inhibits mTOR signaling and activates opposing metabolic pathways. AMPK turns off mTOR by activating the TSC1/TSC2 complex [9] and phosphorylating Raptor, the mTOR binding partner [10]. While mTOR responds to nutrient excess and promotes anabolic processes, AMPK is activated by an increase in the intracellular AMP/ATP ratio (reflective of energy/nutrient depletion) and turns on catabolic processes to restore energy balance [11]. It shuts down gluconeogenesis, lipid synthesis, and protein synthesis while turning on fatty acid oxidation to regenerate the cell’s supply of ATP [12]. Most notably, AMPK accomplishes a shift away from lipid synthesis to fatty acid oxidation by phosphorylating and thereby inactivating acetyl-CoA carboxylase (ACC) [13].

The precise role of AMPK in inflammation is nuanced and an area of active investigation. Although AMPK is not required for T cell development or homeostasis [14,15], AMPK activity is transiently increased post-TCR activation [16]. AMPK is crucial for T cell effector responses [14,17,18] and also regulates memory CD8^+^ T cell development [19] and the recall response [20]. At the same time, AMPK activation can prevent pathological inflammation. AICAR, an activator of AMPK, prevented sepsis in murine models [21] and ameliorated models of ulcerative colitis [22] and multiple sclerosis [23]. Metformin, which acts in part via AMPK activation, was shown to limit inflammation in models of lupus [24] and allograft rejection [25]. By contrast, inhibiting AMPK with compound C exacerbated sepsis in murine models [21].

## 3. Glycolysis

The importance of glycolytic reprogramming in immune activation was one of the earliest observations in immunometabolism, beginning with the discovery that the immune challenge of naïve T cells produces an upregulation of glycolysis critical for T cell effector functions [26,27,28]. This increase in glycolytic flux, with the preferential conversion of pyruvate to lactate rather than oxidation in mitochondria, is akin to the Warburg effect first described in cancer and broadly characterizes the inflammatory response in both adaptive and innate immune cells [29]. As noted above, glycolytic reprogramming critically depends on mTOR activation and the downstream transcription factors HIF-1α and Myc [30,31,32,33].

Although this switch toward aerobic glycolysis has been consistently linked with the differentiation and effector functions of inflammatory cells, glycolytic reprogramming also impacts the function of regulatory cell types. For instance, the upregulation of glycolysis in response to TLR activation or GLUT1 overexpression was shown to increase the proliferation of Treg cells but impair their suppressive functions [34]. More recently, thymus-derived Treg cells were found to increase glycolysis following TNF receptor 2 (TNFR2) stimulation in a manner that enhanced both proliferation and suppressive function, although these cells oxidized pyruvate rather than secreting lactate in a Warburg-like manner [35]. Similarly, although the upregulation of OXPHOS is critically important for the alternative activation of anti-inflammatory M2 macrophages [36], some recent reports using the glycolysis inhibitor 2-deoxyglucose (2-DG) suggested that glycolysis is also required in these cells to support OXPHOS and fatty acid synthesis [37,38,39]. However, a more recent study suggested that glycolysis is dispensable for M2 differentiation, with the inhibitory actions of 2-DG based on off-target effects independent of glycolysis [40].

Several hypotheses are widely held regarding the requirement for glycolytic upregulation following immune activation. For instance, one argument is that increased glycolysis provides critical biomass by supplying precursors necessary for nucleotide, lipid, and protein synthesis. Another argument is that the rapid kinetics of glycolysis support increased bioenergetic requirements by providing more ATP per second despite less efficiency relative to oxidative phosphorylation. Although these hypotheses are biologically plausible, it has become clear that additional mechanisms contribute to the requirement for upregulated glycolysis in inflammatory responses. Thus, while some glycolytic enzymes appear to be key regulators of inflammation simply by controlling metabolic flux, others possess unique and unexpected moonlighting roles or control the supply of metabolites that act not simply as precursors but also as signaling molecules.

**Figure 1:**
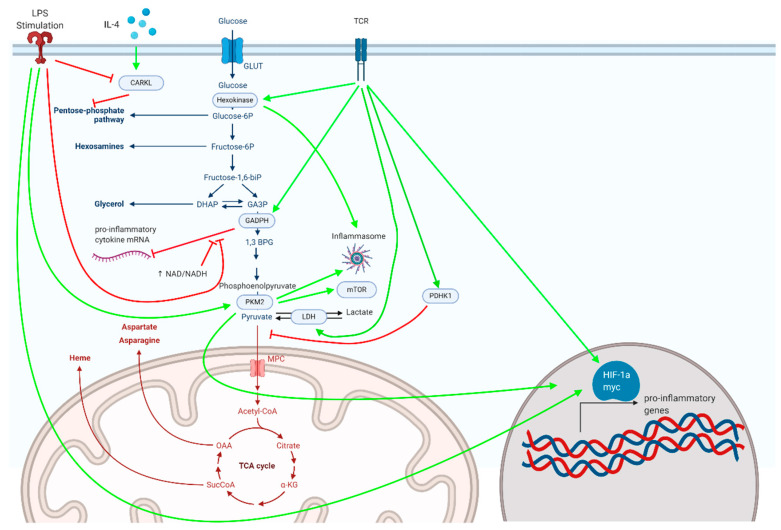
Metabolic enzymes associated with glycolysis and the pentose phosphate pathway, depicted here, serve as critical regulators of inflammatory responses in myeloid and lymphoid cells.

Below, we highlight a number of glycolytic enzymes demonstrated to critically regulate inflammatory responses (Figure 1).

### 3.1. Hexokinase

Hexokinase catalyzes the first step in glycolysis, the conversion of glucose to glucose-6-phosphate. As a critical regulator of glycolytic flux, the transcription of hexokinase, particularly hexokinase 2, is upregulated downstream of TCR and IL-2 receptor signaling. This is achieved by the activation of mTOR and the transcription factors HIF-1α and Myc [3,4,41]. Hexokinase is also particularly important for the HIV infection of macrophages by supporting the survival of HIV-infected cells [42]. The pharmacologic inhibition of hexokinase enzyme activity dampens the inflammatory response. The glycolytic inhibitor 2-DG, which indirectly inhibits hexokinase through the competitive inhibition of the downstream enzyme phosphoglucoisomerase, impairs T cell proliferation and effector functions [27,28]; shifts the balance between effector, regulatory, and memory T cells [31,43]; prevents the pro-inflammatory activation of dendritic cells [44,45,46] and macrophages [47]; and produces benefit in animal models of autoimmunity such as lupus [24] and rheumatoid arthritis (RA) [48]. Furthermore, the direct hexokinase inhibitor 3-bromopyruvate prevents immune activation and attenuates disease in murine models of RA [49] and multiple sclerosis (MS) [50].

Hexokinase enzyme activity plays a somewhat different role in the context of viral infection. In cells infected by a virus, viral RNA is detected by proteins of the retinoic acid-inducible gene 1-like receptor (RLR) family, which in turn trigger the type 1 interferon response through interactions with the mitochondrial antiviral signaling protein (MAVS). Recently, MAVS was shown to interact with hexokinase at the mitochondrial outer membrane, enhancing hexokinase activity [51]. Upon binding to RLR, the MAVS–hexokinase interaction was abolished, producing a decrease in glycolytic flux that was critical for type 1 interferon production.

In addition to its conventional enzymatic function, hexokinase triggers inflammation through a moonlighting role as a pattern recognition receptor. Researchers found that N-acetylglucosamine, a component of peptidoglycan found in bacterial cell walls, inhibits hexokinase [52]. This inhibition causes hexokinase to separate from the mitochondria and activate the NLRP3 inflammasome, leading to the production of pro-inflammatory cytokines. This effect held true when other inhibitors of hexokinase were used.

### 3.2. Glyceraldehyde-3-Phosphate Dehydrogenase (GAPDH)

Glyceraldehyde-3-phosphate dehydrogenase, or GADPH, is another glycolytic enzyme that plays a crucial role in inflammation. As discussed below, GAPDH has a well-defined and highly regulated moonlighting role as an mRNA-binding protein, repressing the translation of inflammatory cytokines in competition with its glycolytic enzyme activity. At the same time, GAPDH appears to play a key role in regulating glycolytic flux under the Warburg conditions that define activated immune cells [53,54].

GADPH binds AU-rich elements within the 3′ untranslated region (3′-UTR) of mRNAs encoding the pro-inflammatory cytokines IFNγ and GM-CSF, repressing their translation. These mRNAs bind GAPDH competitively with NAD^+^/NADH, and the increased glycolytic engagement of GAPDH upon immune activation causes release and increased translation [55,56]. Similarly, in monocytes and macrophages, GADPH represses the translation of TNFα mRNA, which is reversed upon LPS exposure [57]. The relieved repression of cytokine mRNA translation by GAPDH therefore links the upregulation of glycolysis with inflammatory cytokine production.

The moonlighting role of GAPDH as an mRNA-binding protein is regulated by post-translational modification. Malonylation is a lysine modification induced by malonyl-CoA. The malonylation of GADPH occurs downstream of LPS stimulation in monocytes and macrophages, increasing GAPDH enzyme activity while decreasing its mRNA binding capacity, thus allowing inflammatory cytokines such as IFN-γ, IL-6, and TNF-α to be translated into protein [58].

Although not a rate-limiting enzyme under basal conditions, GADPH becomes rate-limiting under Warburg conditions [53,54,59], which may have direct relevance to its role in regulating immune responses. Dimethyl fumarate (DMF), an immunomodulatory drug FDA-approved for the treatment of MS, post-translationally modifies GAPDH at its active site and inactivates its enzymatic activity [60]. The enzymatic inhibition of GAPDH by DMF inhibits glycolysis in activated, but not resting, immune cells and mediates the anti-inflammatory effects of the drug. Interestingly, DMF does not act by altering GAPDH–mRNA binding. Subsequent work demonstrated that itaconate, an anti-inflammatory metabolite derived from the TCA cycle, similarly inactivates GAPDH enzyme activity [61]. These findings suggest that GAPDH enzyme activity is required for pro-inflammatory responses independent of mRNA binding, either by limiting glycolytic flux in a general sense or by regulating the levels of immunoactive metabolites. For instance, the inhibition of GAPDH increases concentrations of methylglyoxal, which has been shown to dampen inflammation by acting on the KEAP1–NRF2 axis [62].

### 3.3. Enolase

Enolase is responsible for the ninth step of glycolysis, converting 2-phosphoglycerate to phosphoenolpyruvate. At the same time, enolase is an abundant protein on bacterial cell surfaces that binds to plasminogen, thus allowing bacteria to invade the host organism [63]. Enolase has been shown to be crucial for the virulence of several strains of bacteria, as evidenced by data showing that inhibiting interaction between enolase and plasminogen [64] or immunizing against enolase prior to challenge with pathogenic bacteria [65] significantly altered the progression of the infection in mouse models. The binding of α-enolase to plasminogen has been shown to be important for the recruitment of macrophages in inflammatory lung disease [66]. Additionally, enolase plays a role in Treg generation through a moonlighting function as a transcriptional regulator. One study found that enolase localizes to the nuclei of T cells to generate Tregs in the periphery. In the nucleus, enolase binds to regulatory regions of *FOXP3* and directly affects the expression of the splicing variant Foxp3-E2, which was corroborated in peripheral blood samples from patients with type 2 diabetes and relapsing-remitting multiple sclerosis [67].

### 3.4. Pyruvate Kinase M2 (PKM2)

Pyruvate kinase (PK) converts phosphoenolpyruvate into pyruvate, a rate-limiting step and the final reaction of glycolysis. Multiple isoforms of PK exist. The M1 isoform (PKM1) is constitutively expressed in most differentiated tissues under basal conditions and exists as a tetramer with high glycolytic activity. The M2 isoform of pyruvate kinase (PKM2) is preferentially expressed under Warburg conditions, such as in cancer cells and activated immune cells, and contributes to the inflammatory response through multiple mechanisms [68]. Unlike PKM1, PKM2 exists either as a tetramer with high glycolytic activity, or as a dimer with low glycolytic activity. Perhaps paradoxically, it is the low-activity PKM2 dimer that promotes aerobic glycolysis and inflammation, largely through non-glycolytic moonlighting functions.

Pro-inflammatory stimuli, such as LPS stimulation of macrophages and TCR ligation in T cells, increase the expression of PKM2 [69,70]. PKM2 in turn activates mTORC1 by phosphorylating the mTOR inhibitor AKT1 substrate 1 (AKT1S1) [71] and increasing serine synthesis from the glycolytic metabolite 3-phosphoglycerate [72]. Despite its intrinsically lower enzyme activity, PKM2 upregulates glycolysis through moonlighting functions as a transcriptional co-activator, promoting the transcriptional program of HIF-1α [73]. HIF-1α increases the expression of PKM2. The dimer form of PKM2 in turn binds to HIF-1α, translocates to the nucleus, and enhances the transcription of HIF-1α target genes.

The non-canonical moonlighting activity of PKM2 as a transcriptional co-activator of HIF-1α is crucial for the inflammatory activation of both macrophages and T lymphocytes. Following the LPS stimulation of macrophages, dimerized nuclear PKM2 drives a pro-inflammatory transcriptional program that includes IL-1β induction. Small molecules such as TEPP-46, which induce the tetramerization of PKM2 and thereby promote its canonical enzyme activity while inhibiting its nuclear functions, inhibit LPS-induced glycolytic reprogramming and inflammatory functions while promoting the expression of anti-inflammatory cytokines such as IL-10 [70]. Similarly, the treatment of CD4^+^ T cells with TEPP-46 blocks PKM2 nuclear translocation, prevents glycolytic reprogramming, and reduces T cell activation, proliferation, and cytokine production [74]. Treatment with TEPP-46 prevents the differentiation of pro-inflammatory Th1 and Th17 cells and attenuates disease in the experimental autoimmune encephalomyelitis (EAE) mouse model of autoimmune neuroinflammation, identifying PKM2 as a potential therapeutic target. Two other groups similarly found that a genetic deficiency of PKM2 protects mice from EAE. One of these demonstrated that the shRNA-mediated knockdown of PKM2 in isolated CD4^+^ cells reduced glycolysis and Th1/Th17 differentiation while also limiting their pathogenicity in an adoptive transfer model of EAE [75]. The other group found that the CD4^+^ T cell-specific knockout of PKM2 impaired Th17 differentiation and attenuated the course of active-immunization EAE, though they observed that PKM2 was required for Th17 differentiation through the activation of the transcription factor STAT3 rather than through actions on HIF-1α and metabolic reprogramming [76]. Similarly, another group found that PKM2 upregulates IL-17 production in CD4^+^ cells via STAT3 in response to lactate uptake [77].

In addition to its nuclear functions, PKM2 has been shown to directly activate the NLRP3 inflammasome in macrophages [78]. AIM2 and NLRP3 inflammasome activation were prevented both by the genetic deletion of PKM2 and a pharmacologic inhibitor of PKM2 enzyme activity, suggesting that its canonical enzyme activity may be important. In natural killer (NK) cells, PKM2 regulates the inflammatory response independent of HIF-1α and its nuclear activities, regulating redox status by controlling the flux of upstream glycolytic metabolites into the pentose phosphate pathway for the generation of NADPH [79]. As such, canonical PKM2 enzyme activity may be important in certain cell types and/or under specific conditions.

### 3.5. Pyruvate Dehydrogenase Kinase 1 (PDHK1)

As the end product of glycolysis, pyruvate has two potential fates—it can either enter the pyruvate dehydrogenase (PDH) complex to become acetyl-CoA and enter the TCA cycle, or be converted to lactate by lactate dehydrogenase (LDH). When pyruvate is reduced to lactate, it regenerates NAD^+^, thus allowing glycolysis and the Warburg phenotype to continue. In both CD4^+^ and CD8^+^ T cells, TCR stimulation increases the expression and activity of pyruvate dehydrogenase kinase 1 (PDHK1), a kinase that inhibits PDH and thereby diverts pyruvate away from the TCA cycle and toward lactate production [80,81]. PDHK1 activity is high in pro-inflammatory Th17 cells but low in Treg cells. The inhibition of PDHK1 with the small molecule dichloroacetate (DCA) limits aerobic glycolysis and promotes pyruvate entry into the TCA cycle, thereby limiting Th17 and augmenting Treg generation from naïve CD4^+^ cells and inhibiting inflammatory cytokine production in CD8^+^ cells. Treatment with DCA in vivo produces benefits in animal models of multiple autoimmune diseases, including inflammatory bowel disease, RA, MS, and asthma [81,82,83]. These findings implicate PDHK1 not only as a key regulator of aerobic glycolysis and inflammation, but also as a potential therapeutic target in autoimmunity.

### 3.6. Lactate Dehydrogenase (LDH)

As noted above, the conversion of pyruvate to lactate by LDH regenerates NAD^+^ and maintains high glycolytic flux under Warburg conditions. Through several mechanisms, LDH critically regulates cellular inflammatory responses. The pro-inflammatory activation of CD4^+^ cells leads to increased expression of LDH-A, an isoform of LDH with high enzymatic activity. LDH-A activity promotes the inflammatory response by maintaining high levels of acetyl-CoA, which in turn promotes histone acetylation and the transcription of IFNγ [84]. LDH-A-deficient mice were protected from autoimmune attacks. The inhibition of LDH with the small molecule FX11 (3-dihydroxy-6-methyl-7-(phenylmethyl)-4-propylnaphthalene-1-carboxylic acid) has been shown to inhibit the release of pro-inflammatory cytokines in macrophages [85]. Similar to GAPDH, LDH has also been found to bind and repress the translation of mRNAs encoding inflammatory cytokines, releasing them upon the engagement of its enzymatic activity [80,86]. Finally, lactate itself has been shown to play a direct signaling role in inflammation. In CD4^+^ cells, lactate uptake via the transporter SLC5A12 was shown to promote Th17 differentiation and prevent migratory egress from sites of inflammation, and the blockade of lactate uptake attenuated disease in a model of autoimmune arthritis [77]. In many other studies, however, lactate has been found to produce anti-inflammatory effects in both lymphocytes and macrophages. Earlier studies demonstrated that lactate produced in the tumor microenvironment (TME) has suppressive effects on effector and cytotoxic T cells [87,88]. By contrast, it was recently reported that Foxp3 induces metabolic changes in Treg cells that allow them to survive and function in a low-glucose, high-lactate TME [89]. Another study found that lactate specifically promotes Foxp3 expression [90]. Through a variety of mechanisms, extracellular lactate similarly induces regulatory phenotypes in tumor-associated macrophages, contributing to tumor evasion [91,92,93]. More recently, lactate was found to produce a post-translational modification of histones (lactylation) that regulates transcription and shifts cells toward an anti-inflammatory M2 phenotype as a late event following LPS stimulation [94]. Interestingly, in the context of viral infection, lactate dampens type 1 interferon production by infected cells through a direct interaction with MAVS [51]. As such, mice deficient in LDH-A displayed a greater type 1 interferon response and heightened resistance to infection with vesicular stomatitis virus.

## 4. Mitochondrial Metabolism—TCA Cycle and Electron Transport Chain

Although glycolytic reprogramming represents a common theme among pro-inflammatory immune cells, mitochondrial metabolism also plays a vital role in inflammatory responses. The activation of naïve T cells leads to increased oxidative phosphorylation (OXPHOS) [95], and memory lymphocytes generate modified mitochondrial networks that allow them to better carry out the TCA cycle and OXPHOS [96]. By contrast, macrophages and dendritic cells experience a drastic reduction in OXPHOS upon inflammatory activation, yet this disruption of the TCA cycle and electron transport chain (ETC) nonetheless plays a key role in inflammation by providing metabolites and reactive oxygen species (ROS) that serve as signaling molecules [47,95,97]. Oxidative metabolism skews toward an anti-inflammatory phenotype in myeloid cells [36], as further evidenced by the fact that monocytes found within a *Staphylococcus aureus* biofilm primarily used OXPHOS, while OXPHOS inhibition via the nanoparticle delivery of oligomycin skewed cells toward a glycolytic phenotype and induced bacterial clearance [98].

In this section, we will focus on key enzymes within the TCA cycle and ETC that regulate inflammatory responses.

### 4.1. TCA Cycle

Fed by glucose, protein, and fatty acids, the TCA cycle generates electron donors (NADH and FADH_2_) to support OXPHOS through the ETC. However, in addition to its role in energy generation, the TCA cycle produces metabolites with key roles in inflammation. For instance, the inflammatory activation of macrophages leads to increased levels of citrate, succinate, and itaconate, each of which plays specific roles in the immune response [97]. While a comprehensive discussion of the roles of these metabolites is beyond our scope, we will highlight key enzyme targets that regulate the availability of these metabolites or their downstream effects relevant to inflammation.

#### 4.1.1. Isocitrate Dehydrogenase (IDH)

The inflammatory activation of macrophages leads to a decreased activity of isocitrate dehydrogenase (IDH), potentially via both transcriptional repression [47] and inactivation by nitric oxide (NO)-mediated S-nitrosylation [99]. This “break” in the TCA cycle leads to the accumulation of citrate and itaconate, although it must be noted that aconitase, rather than IDH, has recently been implicated as the target of NO mediating this break [100]. As discussed below, accumulated citrate can be converted to itaconate within mitochondria or be transported to the cytosol. Within the cytosol, citrate serves several functions regulated by key enzymes. ATP-citrate lyase (ACLY), which converts citrate to oxaloacetate and acetyl-CoA, contributes to NO and ROS production through an unknown mechanism [101] and generates a pool of acetyl-CoA serving histone acetylation [102], lipogenesis [103], and malonylation [104].

#### 4.1.2. Immune-Responsive Gene 1 Protein (IRG1)

Immune-responsive gene 1 (IRG1) is responsible for catalyzing the conversion of cis-aconitate (an intermediate of the TCA cycle) into itaconic acid [105]. The transcription of IRG1 is upregulated in the pro-inflammatory state in macrophages as a response to various stimuli, such as IFNγ, LPS, and TNFα [106]. During inflammation, IRG1 produces itaconate, which has a direct bactericidal role by altering bacterial metabolism [107]. However, other studies have shown that itaconate has anti-inflammatory effects via multiple mechanisms [105,108], such as the inhibition of succinate dehydrogenase (SDH) [109] and post-translational modification of key protein targets such as KEAP1 [110] and GAPDH [61].

#### 4.1.3. Succinate Dehydrogenase (SDH)

SDH is a key enzyme in the TCA cycle, converting succinate into fumarate [97]. SDH plays a role in generating a pro-inflammatory phenotype by contributing to the production of pro-inflammatory cytokines such as IL-1β and reactive oxygen species (ROS) in macrophages in vitro; the inhibition of SDH produces anti-inflammatory effects, as discussed above in the context of itaconate [109,111].

### 4.2. Electron Transport Chain (ETC) and ROS

The generation of ATP from OXPHOS is mediated by the ETC, which accepts electrons from NADH and FADH_2_ produced from the TCA cycle and generates ATP via ATP synthase. Because OXPHOS increases in lymphocytes after activation, the electron transport chain is required for lymphocytes to be properly activated, and ATP synthase activity is one of the most important parts of this process [112]. When complex IV is knocked out, T cell activation is inhibited [112], while complex III deficiency prevents T cell proliferation in vivo and in vitro [113]. At the same time, bacterial RNA stimulates complex II activity in macrophages, and the inhibition of complex II with a small molecule inhibitor significantly increased death rates due to increasing rates of sepsis and the decreased release of pro-inflammatory cytokines when mice were infected with *Salmonella enterica* [114]. Furthermore, HIF-1α and IL-1β are linked to the production of NO, which can turn off the ETC in macrophages [115]. The ETC is also the target of novel pharmacological interventions for autoimmunity, such as LYC-30937-ec, an F_1_F_0_ ATP synthesis inhibitor, which is in clinical trials for ulcerative colitis [116].

The upregulation of OXPHOS plays an important role in the alternative activation of macrophages in response to signals such as IL-4 [36]. An important regulator of OXPHOS in this context is the polyamine–eIF5A–hypusine axis [117]. Hypusine, a natural amino acid derived from the polyamine spermidine [118], which itself is a downstream metabolite of arginine, post-translationally modifies the translation factor eIF5A [117]. This increases the expression of several enzymes involved in the TCA cycle and OXPHOS. The genetic or pharmacologic inhibition of key enzymes mediating eIF5a hypusination, namely, deoxyhypusine synthase (DHPS) and deoxyhypusine hydroxylase (DOHH), blunts OXPHOS-dependent alternative macrophage activation [117].

Although the ETC is important in generating ATP for the cell, it is also a primary source of ROS [119]. ROS induce inflammation in the cell in a variety of ways, including the activation of the NLRP3 inflammasome [95]. LPS stimulation induces ROS production, which plays a direct role in the bactericidal activity of the cell but also induces HIF-1α expression and the subsequent metabolic reprogramming downstream of HIF-1α [95]. Mitochondrial ROS (mROS) generation from complex III is required for macrophage activation and stimulates the NAD^+^ salvage pathway [120]. Furthermore, mROS are crucial for T cell function. For example, complex III-deficient T cells are unable to produce IL-2, but treatment with exogenous ROS in the form of H_2_O_2_ reverses this effect [113]. A high-glucose diet exacerbates autoimmunity in mouse models by promoting the differentiation of Th17 cells, which depends on mROS-induced TGFβ activation [121]. Further demonstrating the importance of ROS in inflammation, pre-treating cells with antioxidants inhibits the production of ROS and thus inhibits the release of pro-inflammatory cytokines [113,120,121].

Several pharmacologic treatments target ROS generation. For example, metformin is a complex I inhibitor and thereby limits the production of mROS [1]. Although metformin is most well known as a type II diabetes drug, data also suggest it exerts anti-inflammatory effects. It has been shown to decrease the production of anti-inflammatory cytokines, as well as limiting the production of HIF-1α and the activity of mTORC1 [122]. Metformin largely exerts its inhibitory effect on complex I by activating AMPK, and the developers of several other drugs seek to utilize this effect through the development of novel AMPK activators as potential therapeutics for autoimmune diseases [123].

## 5. The Pentose Phosphate Pathway

The pentose phosphate pathway (PPP) is a critical metabolic process that is responsible for nucleotide synthesis and the generation of NADPH. While nucleotide synthesis is vital for the mitosis of rapidly proliferating cells, NADPH is a critical regulator of cellular redox status and fatty acid synthesis [124]. In the context of infection, NADPH is important for generating the ROS used by activated neutrophils and macrophages to clear bacterial infections. To generate these critical products, the PPP is upregulated during inflammation, especially in M1 macrophages [125].

### CARKL

The PPP is regulated during inflammatory processes through an enzyme known as CARKL (Figure 1). CARKL is a sudoheptulose kinase that catalyzes an orphan reaction that downregulates the non-oxidative arm of the PPP, and its expression dictates macrophage activation and polarization. LPS downregulates while the anti-inflammatory cytokine IL-4 upregulates CARKL, and macrophage polarization can be altered by the genetic manipulation of CARKL [126]. Therefore, by responding to pro- or anti-inflammatory signals, CARKL regulates the PPP and hence shifts macrophages toward an appropriate metabolic phenotype. Much like how CARKL can dampen the inflammatory response by inhibiting the PPP, researchers are also designing novel therapeutics that will target this pathway. For instance, RRx-001 is an inhibitor of glucose-6-phosphate dehydrogenase that has been shown to have potent anti-inflammatory effects in vitro and is currently in clinical trials [127].

## 6. Fatty Acid Metabolism

**Figure 2:**
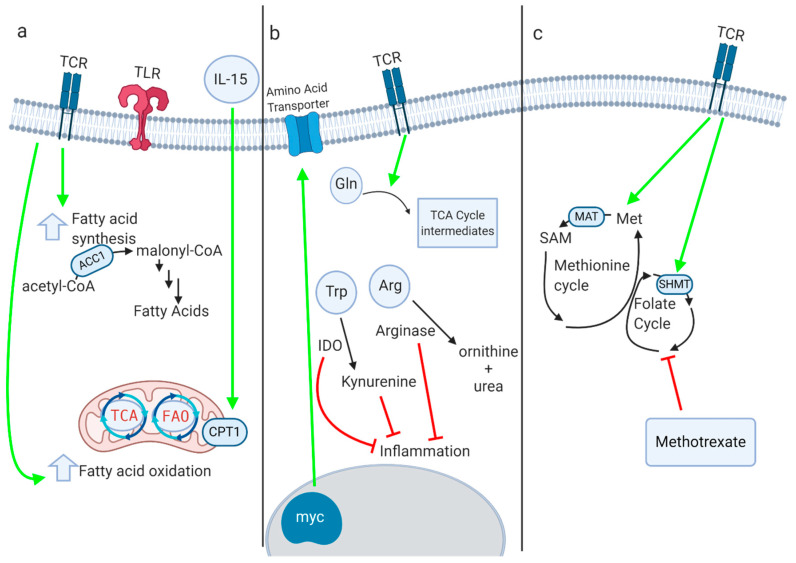
(**a**) Fatty acid oxidation (FAO) and fatty acid synthesis both play key roles in myeloid and lymphoid immune responses. Several enzymes within these pathways, such as CPT1a and ACC1, critically regulate immune phenotype and function, although the role of CPT1a in T cells has recently been questioned. (**b**) Immune activation leads to increased amino acid transport and glutaminolysis. Enzymes such as IDO and arginase-1 inhibit inflammation by depleting amino acids and producing anti-inflammatory immunoactive metabolites. (**c**) One-carbon metabolism supports inflammation through a variety of mechanisms. Inhibition of one-carbon metabolism by inactivation of MAT2a or SHMT suppresses inflammatory responses. Methotrexate, a dihydrofolate reductase inhibitor that interferes with one-carbon metabolism, is one of the oldest immunosuppressive drugs in clinical use.

Fatty acid oxidation (FAO) breaks down fatty acids to generate acetyl-CoA, which enters the TCA cycle to drive ATP synthesis through OXPHOS. Treg and CD8^+^ memory T cells utilize copious amounts of FAO [128]. Following CD4^+^ cell activation, increasing FAO with an AMPK activator drove a Treg phenotype [128]. At the same time, FAO is important for a memory phenotype, with IL-15, a cytokine that is critical for the generation of CD8^+^ memory T cells, promoting FAO by increasing mitochondrial biogenesis and increasing the expression of carnitine palmitoyl transferase (CPT1a), the rate-limiting step of mitochondrial long-chain fatty acid oxidation (FAO) [129] (Figure 2). It should be noted, however, that a recent report employing a genetic deficiency of CPT1a suggested that the enzyme is dispensable for T cell activation and the generation of memory CD8^+^ and Treg cells, and that AMPK activation augments Treg differentiation independent of CPT1a [130]. Other studies have found that TNF receptor-associated factor 6 (TRAF6), an adaptor protein in the TNF-receptor superfamily, is also critical for CD8^+^ memory T cell generation [19]. Additionally, one of the most important mechanisms for upregulating FAO in memory T cells involves mobilizing fatty acids through lysosomal hydrolase LAL (lysosomal acid lipase) [131].

In macrophages, M1 macrophages primarily utilize glycolysis, while M2 macrophages heavily rely on FAO. Overexpressing CPT1a, the rate limiting factor in long-chain FAO, in macrophages in vitro reduced the release of pro-inflammatory cytokines and promoted M2 polarization [132]. Researchers have sought to therapeutically target this pathway with the purported CPT1a inhibitor etomoxir, which mitigated MS symptoms in the EAE mouse model [133] and prevented graft versus host disease in a murine model [134]. However, etomoxir has recently been demonstrated to have off-target immunomodulatory effects beyond CPT1a, which were suggested to be the reason for discrepancies between studies employing genetic and pharmacologic inhibition to study the role of CPT1a in T cell function [130].

Fatty acid synthesis also plays a crucial role in inflammation. In dendritic cells (DCs), TLR activation upregulates fatty acid synthesis, which supports the expansion of the endoplasmic reticulum and Golgi and is required for DC activation [135]. Fatty acid synthesis is also required for proper T cell activation [136]. In T cells, the ratio of cholesterol in the cell membrane is important for proper activation, and inhibiting the cholesterol esterification enzyme acetyl-CoA acetyltransferase (ACAT1) generated a more robust effector response [137]. Acetyl-CoA carboxylase 1 (ACC1) is the rate-limiting factor in fatty acid synthesis, and T cells deficient in ACC1 had impaired proliferation and effector capacity [138] (Figure 2). Interestingly, ACC1 deficiency does not affect Treg function and instead promotes Treg differentiation over Th17 polarization [139]. Fatty acid synthesis can be pharmacologically targeted with the ACC1 inhibitor soraphen A, which alleviated disease severity and symptom onset in EAE by inhibiting Th17 polarization.

## 7. One-Carbon Metabolism

One-carbon metabolism involves the transfer of one-carbon (-CH_3_) units within the cell and is critical for multiple biosynthetic processes, including the generation of nucleotides, amino acids, and fatty acids, and for epigenetic regulation via methylation. One-carbon units incorporated into this pathway can be derived from folate metabolism or donated from amino acids such as serine, glycine, or methionine [140]. Activated immune cells have an increased requirement for one-carbon metabolism [141,142,143]. TCR stimulation upregulates one-carbon metabolism [144], while in macrophages, LPS stimulation activates both the serine synthesis pathway and one-carbon metabolism in order to drive epigenetic changes required for the release of pro-inflammatory cytokines such as IL-1β [145,146]. In general, one-carbon metabolism supports immune activation and proliferation/survival through multiple mechanisms, including providing biosynthetic precursors for anabolic processes [142,143], regulating redox status by controlling glutathione levels [146,147] and providing the substrate for histone methylation (S-adenosylmethionine or SAM) [145].

Several key enzymes involved in one-carbon metabolism have been shown to regulate inflammation (Figure 2). In macrophages, the genetic or pharmacologic inhibition of methionine adenosyl transferase (MAT2a) led to decreased IL-1β production, as did treatment with 3-deazaadenosine, an inhibitor of SAH hydroxylase [145]. The inhibition of serine hydroxymethyl transferase 1 and 2 (SHMT1 and SHMT2), which feed one-carbon metabolism through serine, decreased the production of inflammatory cytokines and protected mice from LPS-induced sepsis. The pharmacologic and genetic inhibition of SHMT isoforms similarly inhibited T cell proliferation [142,143].

No discussion of one-carbon metabolism would be complete without mentioning methotrexate, which is one of the most widely used and oldest anti-inflammatory drugs on the market. Methotrexate is a dihydrofolate reductase inhibitor, thus inhibiting folate metabolism, one-carbon metabolism, and nucleotide synthesis. However, when used as a treatment for autoimmune diseases, its precise mechanism of action is controversial, with some evidence suggesting it acts via the release of extracellular adenosine and activation of AMPK [148]. Nevertheless, other novel therapeutics targeting this pathway show promise in pre-clinical models of cancer or autoimmunity [141].

## 8. Amino Acid Metabolism

Protein metabolism and amino acid availability are critical regulators of the immune response (Figure 2). Following T cell activation, upregulated Myc expression drives the upregulation of amino acid transporters so that they can shuttle amino acids into the cell [30]. Both leucine and glutamine transporters are upregulated after T cell activation, and the genetic deletion of these transporters inhibits the T cell response [149,150]. Furthermore, the availability of amino acids in the extracellular environment is relevant for T cell expansion. For example, glutamine is included in excess in cell culture media, and restricting glutamine will restrict T cell expansion in vitro. In cancer, myeloid-derived suppressor cells inhibit T cell activation by depleting cystine and cysteine in the tumor microenvironment [151]. As noted above, serine availability, derived both from exogenous pools and de novo synthesis, critically regulates T cell and macrophage activation [142,143,145,146].

There are several enzymes that play a key role in regulating amino acid availability to control the immune response. Indoleamine 2,3-dioxygenase (IDO) depletes tryptophan, thereby inhibiting T cell responses [152]. IDO is expressed at high levels in the placenta to prevent T cell activation and promote feto-maternal tolerance [153]. Kynurenine, the downstream metabolite of IDO, is also immunosuppressive, and cancer researchers are seeking to therapeutically alter this pathway [154]. Tryptophan metabolism via the kynurenine pathway also regulates macrophage function by controlling de novo NAD^+^ synthesis [155]. The genetic or pharmacologic inhibition of quinolate phosphoribosyltransferase (QPRT), which provides the NAD^+^ precursor nicotinic acid mononucleotide (NaMN), augments the pro-inflammatory response to LPS. Conversely, the ectopic expression of QPRT dampens inflammation and promotes a homeostatic macrophage phenotype. Arginase-1 is preferentially expressed in M2 macrophages, and this enzyme depletes arginine, hence preventing the formation of the pro-inflammatory nitric oxide generated by M1 macrophages [156,157]. During infection with *Mycobacterium tuberculosis*, macrophages in the lung granulomas pathognomonic for the disease produce high levels of Arg1 to control the inflammatory response [158].

Glutaminolysis is the process by which the cell breaks down glutamine and converts it into TCA cycle intermediates and other metabolites. In activated T cells, glutaminolysis is upregulated in a Myc-dependent manner; this upregulation of glutamine catabolism replenishes TCA cycle intermediates, fuels polyamine synthesis, and coordinates with glucose catabolism to support amino acid, nucleotide, and lipid biosynthesis [30]. Moreover, glutamate oxaloacetate transaminase 1 (GOT1), an enzyme involved in glutamine metabolism, exerts pro-inflammatory effects by producing 2-hydroxyglutarate, which hinders the expression of FOXP3 and thus blocks the formation of Tregs. Researchers have tried inhibiting glutamine metabolism with the glutamine analog 6-diazo-5-oxo-L-norleucine (DON), and this molecule suppressed inflammation in mouse models of acute lung injury [159] and prevented allograft rejection [25]. Although inhibiting glutamine metabolism in models of autoimmunity alleviates the autoimmune phenotype, in cancer models, inhibiting glutamine metabolism generates a more potent anti-tumor immune response. Researchers found that in several cancer models, inhibiting glutamine metabolism with DON or a related prodrug generated effector CD8^+^ T cells capable of a robust anti-tumor response. Glutamine antagonism selectively upregulated OXPHOS in tumor-infiltrating CD8^+^ cells via an increased activity of acyl-coenzyme A (CoA) synthetase short-chain family member 1 (ACSS1), allowing the fueling of the TCA cycle through acetate [160].

## 9. Autophagy

Autophagy is the metabolic process by which cells degrade and recycle cellular components. Autophagy has been shown to be important for immune cell activation; the knockout of the autophagy-essential gene Atg3 resulted in apoptosis and inhibited the activation of murine immune cells in vitro [161]. This study also showed that hematopoietic cells use autophagy to provide lipids to the cell when they become metabolically stressed. Following TCR or TLR activation, immune cells upregulate autophagy to generate the nutrients needed for the metabolic demands of the cell [162]. On the other hand, autophagy also plays a role in inducing cell death during HIV infection, with the HIV glycoprotein gp120 binding to CXCR4 on T cells and inducing apoptosis, but this effect was abrogated by the deletion of autophagy-critical enzymes such as Beclin-1 or Atg7 [163]. Autophagy also plays a major role in immune cell differentiation [164]. Autophagy is turned off by mTOR and turned on by AMPK, which also allows the cell to regulate Treg and memory T cell fate by using autophagy to generate the lipids needed for the FAO-dependent metabolic phenotype of these cells [164]. Autophagy also controls inflammatory responses in myeloid cells; the deletion of autophagy-critical genes promoted an M1 inflammatory phenotype and inhibited M2 polarization [165]. Autophagy is also relevant for the neutrophil response to infection, as it upregulates cell-intrinsic survival mechanisms [166].

## 10. Conclusions

Immunometabolism is a rapidly burgeoning field, with many complex and interconnected metabolic pathways now understood to influence immune activation and inflammation. Within these complex networks, we have sought to highlight key metabolic enzymes that serve as critical regulators of inflammation and their potential for pharmacologic modulation. With various drugs targeting these enzymes in pre-clinical or clinical trials, and in some cases already in clinical use (Table 1), modulating inflammation by targeting metabolic enzymes has the potential to take the field of immunometabolism from bench to bedside in the treatment of inflammatory diseases.

## Figures and Tables

**Table 1:** A sample of potential and currently used therapeutics targeting metabolic enzymes to modulate inflammation.

Enzymatic Target	Therapeutic Compound	Disease	Trial Status
mTOR [2,3,4,5]	Rapamycin	Organ Transplant	FDA-approved drug
Hexokinase [24,48,49,50]	3-bromopyruvate	Rheumatoid Arthritis, Multiple Sclerosis	Pre-clinical
GADPH [60]	Dimethyl fumarate	Multiple Sclerosis	FDA-approved drug
Pyruvate kinase M2 [70,74,75]	TEPP-46	Multiple Sclerosis	Pre-clinical
Pyruvate dehydrogenase kinase 1 [81,82,83]	Dichloroacetate	Rheumatoid Arthritis, Inflammatory Bowel Disease, MS, Asthma	Pre-clinical
Lactate dehydrogenase [85]	FX11	Research ongoing	Pre-clinical
Complex I of the electron transport chain (ETC)	Metformin	Diabetes, others ongoing	FDA-approved drug
F_1_F_0_ ATP synthase [116]	LYC-30937-ec	Ulcerative Colitis	Clinical trials
ACC1 [139]	Soraphen A	Multiple Sclerosis	Pre-clinical
Dihydrofolate reductase [141]	Methotrexate	Rheumatoid Arthritis	FDA-approved drug
Glutamine metabolism [25,159,160]	6-diazo-5-oxo-L-norleucine (DON) and prodrug JHU083	Acute Lung Injury, Allograft Rejection, Anti-Tumor Response	Pre-clinical

## References

[1] Patel C.H., Leone R.D., Horton M.R., Powell J.D. (2019). Targeting metabolism to regulate immune responses in autoimmunity and cancer. Nat. Rev. Drug Discov..

[2] Hay N., Sonenberg N. (2004). Upstream and downstream of mTOR. Genes Dev..

[3] Waickman A.T., Powell J.D. (2012). mTOR, metabolism, and the regulation of T-cell differentiation and function. Immunol. Rev..

[4] Weichhart T., Hengstschläger M., Linke M. (2015). Regulation of innate immune cell function by mTOR. Nat. Rev. Immunol..

[5] Saunders R.N., Metcalfe M.S., Nicholson M.L. (2001). Rapamycin in transplantation: A review of the evidence. Kidney Int..

[6] Delgoffe G.M., Kole T.P., Zheng Y., Zarek P.E., Matthews K.L., Xiao B., Powell J.D. (2009). The mTOR Kinase Differentially Regulates Effector and Regulatory T Cell Lineage Commitment. Immunity.

[7] Delgoffe G.M., Pollizzi K.N., Waickman A.T., Heikamp E., Meyers D.J., Horton M.R., Powell J.D. (2011). The kinase mTOR regulates the differentiation of helper T cells through the selective activation of signaling by mTORC1 and mTORC2. Nat. Immunol..

[8] Lee K., Gudapati P., Dragovic S., Spencer C., Joyce S., Killeen N., Boothby M. (2010). Mammalian target of rapamycin protein complex 2 regulates differentiation of Th1 and Th2 cell subsets via distinct signaling pathways. Immunity.

[9] Inoki K., Zhu T., Guan K.L. (2003). TSC2 mediates cellular energy response to control cell growth and survival. Cell.

[10] Gwinn D.M., Shackelford D.B., Egan D.F., Mihaylova M.M., Mery A., Vasquez D.S., Turk B.E., Shaw R.J. (2008). AMPK phosphorylation of raptor mediates a metabolic checkpoint. Mol. Cell.

[11] Hardie D.G. (2011). AMP-activated protein kinase-an energy sensor that regulates all aspects of cell function. Genes Dev..

[12] Herzig S., Shaw R.J. (2018). AMPK: Guardian of metabolism and mitochondrial homeostasis. Nat. Rev. Mol. Cell Biol..

[13] Davies S.P., Sim A.T.R., Hardie D.G. (1990). Location and function of three sites phosphorylated on rat acetyl-CoA carboxylase by the AMP-activated protein kinase. Eur. J. Biochem..

[14] Blagih J., Coulombe F., Vincent E.E., Dupuy F., Galicia-Vázquez G., Yurchenko E., Raissi T.C., vanderWindt G.J.W., Viollet B., Pearce E.L. (2015). The energy sensor AMPK regulates T Cell metabolic adaptation and effector responses invivo. Immunity.

[15] Mayer A., Denanglaire S., Viollet B., Leo O., Andris F. (2008). AMP-activated protein kinase regulates lymphocyte responses to metabolic stress but is largely dispensable for immune cell development and function. Eur. J. Immunol..

[16] Tamás P., Hawley S.A., Clarke R.G., Mustard K.J., Green K., Hardie D.G., Cantrell D.A. (2006). Regulation of the energy sensor AMP-activated protein kinase by antigen receptor and Ca2+ in T lymphocytes. J. Exp. Med..

[17] Ma E.H., Poffenberger M.C., Wong A.H.T., Jones R.G. (2017). The role of AMPK in T cell metabolism and function. Curr. Opin. Immunol..

[18] Rao E., Zhang Y., Zhu G., Hao J., Persson X.M.T., Egilmez N.K., Suttles J., Li B. (2015). Deficiency of AMPK in CD8+ T cells suppresses their anti-tumor function by inducing protein phosphatase-mediated cell death. Oncotarget.

[19] Pearce E.L., Walsh M.C., Cejas P.J., Harms G.M., Shen H., Wang L.S., Jones R.G., Choi Y. (2009). Enhancing CD8 T-cell memory by modulating fatty acid metabolism. Nature.

[20] Rolf J., Zarrouk M., Finlay D.K., Foretz M., Viollet B., Cantrell D.A. (2013). AMPKα1: A glucose sensor that controls CD8 T-cell memory. Eur. J. Immunol..

[21] Escobar D.A., Botero-Quintero A.M., Kautza B.C., Luciano J., Loughran P., Darwiche S., Rosengart M.R., Zuckerbraun B.S., Gomez H. (2015). Adenosine monophosphate-activated protein kinase activation protects against sepsis-induced organ injury and inflammation. J. Surg. Res..

[22] Bai A., Ma A.G., Yong M., Weiss C.R., Ma Y., Guan Q., Bernstein C.N., Peng Z. (2010). AMPK agonist downregulates innate and adaptive immune responses in TNBS-induced murine acute and relapsing colitis. Biochem. Pharmacol..

[23] Paintlia A.S., Paintlia M.K., Singh I., Singh A.K. (2006). Immunomodulatory effect of combination therapy with lovastatin and 5-aminoimidazole-4-carboxamide-1-β-D-ribofuranoside alleviates neurodegeneration in experimental autoimmune encephalomyelitis. Am. J. Pathol..

[24] Yin Y., Choi S.C., Xu Z., Perry D.J., Seay H., Croker B.P., Morel L. (2015). Normalization of CD4+ T cell metabolism reverses lupus. Sci. Transl. Med..

[25] Lee C.F., Lo Y.C., Cheng C.H., Furtmüller G.J., Oh B., Andrade-Oliveira V., Thomas A.G., Bowman C.E., Slusher B.S., Wolfgang M.J. (2015). Preventing allograft rejection by targeting immune metabolism. Cell Rep..

[26] Frauwirth K.A., Riley J.L., Harris M.H., Parry R.V., Rathmell J.C., Plas D.R., Elstrom R.L., June C.H., Thompson C.B. (2002). The CD28 signaling pathway regulates glucose metabolism. Immunity.

[27] Cham C.M., Driessens G., O’Keefe J.P., Gajewski T.F. (2008). Glucose deprivation inhibits multiple key gene expression events and effector functions in CD8+ T cells. Eur. J. Immunol..

[28] Cham C.M., Gajewski T.F. (2005). Glucose Availability regulates IFN-γ production and p70S6 Kinase activation in CD8 + effector T Cells. J. Immunol..

[29] Kornberg M.D. (2020). The immunologic Warburg effect: Evidence and therapeutic opportunities in autoimmunity. Wiley Interdiscip. Rev. Syst. Biol. Med..

[30] Wang R., Dillon C.P., Shi L.Z., Milasta S., Carter R., Finkelstein D., McCormick L.L., Fitzgerald P., Chi H., Munger J. (2011). The transcription factor myc controls metabolic reprogramming upon T lymphocyte activation. Immunity.

[31] Shi L.Z., Wang R., Huang G., Vogel P., Neale G., Green D.R., Chi H. (2011). HIF1α-dependent glycolytic pathway orchestrates a metabolic checkpoint for the differentiation of TH17 and Treg cells. J. Exp. Med..

[32] Byles V., Covarrubias A.J., Ben-Sahra I., Lamming D.W., Sabatini D.M., Manning B.D., Horng T. (2013). The TSC-mTOR pathway regulates macrophage polarization. Nat. Commun..

[33] Saeed S., Quintin J., Kerstens H.H.D., Rao N.A., Aghajanirefah A., Matarese F., Stunnenberg H.G. (2014). Epigenetic programming of monocyte-to-macrophage differentiation and trained innate immunity. Science.

[34] Gerriets V.A., Kishton R.J., Johnson M.O., Cohen S., Siska P.J., Nichols A.G., Rathmell J.C. (2016). Foxp3 and toll-like receptor signaling balance T reg cell anabolic metabolism for suppression. Nat. Immunol..

[35] de Kivit S., Mensink M., Hoekstra A.T. (2020). Stable human regulatory T cells switch to glycolysis following TNF receptor 2 costimulation. Nat. Metab..

[36] Vats D., Mukundan L., Odegaard J.I., Zhang L., Smith K.L., Morel C.R., Chawla A. (2006). Oxidative metabolism and PGC-1β attenuate macrophage-mediated inflammation. Cell Metab..

[37] Covarrubias A.J., Aksoylar H.I., Yu J. (2016). Akt-mTORC1 signaling regulates Acly to integrate metabolic input to control of macrophage activation. eLife.

[38] Huang S.C.C., Smith A.M., Everts B. (2016). Metabolic reprogramming mediated by the mTORC2-IRF4 signaling axis is essential for macrophage alternative activation. Immunity.

[39] Zhao Q., Chu Z., Zhu L. (2017). 2-Deoxy-d-glucose treatment decreases anti-inflammatory M2 macrophage polarization in mice with tumor and allergic airway inflammation. Front. Immunol..

[40] Wang F., Zhang S., Vuckovic I. (2018). Glycolytic Stimulation Is Not a Requirement for M2 Macrophage Differentiation. Cell Metab..

[41] Gnanaprakasam J.N.R., Wang R. (2017). MYC in regulating immunity: Metabolism and beyond. Genes.

[42] Sen S., Kaminiski R., Deshmane S., Langford D., Khalili K., Amini S., Datta P.K. (2015). Role of hexokinase-1 in the survival of hiv-1-infected macrophages. Cell Cycle.

[43] Sukumar M., Liu J., Ji Y., Subramanian M., Crompton J.G., Yu Z., Roychoudhuri R., Palmer D.C., Muranski P., Karoly E.D. (2013). Inhibiting glycolytic metabolism enhances CD8+ T cell memory and antitumor function. J. Clin. Investig..

[44] Everts B., Pearce E.J. (2014). Metabolic control of dendritic cell activation and function: Recent advances and clinical implications. Front. Immunol..

[45] Everts B., Amiel E., van der Windt G.J.W., Freitas T.C., Chott R., Yarasheski K.E., Pearce E.L., Pearce E.J. (2012). Commitment to glycolysis sustains survival of NO-producing inflammatory dendritic cells. Blood.

[46] Krawczyk C.M., Holowka T., Sun J., Blagih J., Amiel E., DeBerardinis R.J., Cross J.R., Jung E., Thompson C.B., Jones R.G. (2010). Toll-like receptor-induced changes in glycolytic metabolism regulate dendritic cell activation. Blood.

[47] Tannahill G. (2013). Succinate is an inflammatory signal that induces IL-1beta through HIF-1alpha. Nature.

[48] Abboud G., Choi S.C., Kanda N., Zeumer-Spataro L., Roopenian D.C., Morel L. (2018). Inhibition of glycolysis reduces disease severity in an autoimmune model of rheumatoid arthritis. Front. Immunol..

[49] Okano T., Saegusa J., Nishimura K., Takahashi S., Sendo S., Ueda Y., Morinobu A. (2017). 3-bromopyruvate ameliorate autoimmune arthritis by modulating Th17/Treg cell differentiation and suppressing dendritic cell activation. Sci. Rep..

[50] Seki S.M., Stevenson M., Rosen A.M., Arandjelovic S., Gemta L., Bullock T.N.J., Gaultier A. (2017). Lineage-specific metabolic properties and vulnerabilities of T Cells in the demyelinating central nervous system. J. Immunol..

[51] Zhang W., Wang G., Xu Z.G., Tu H., Hu F., Dai J., Lin H.K. (2019). Lactate is a natural suppressor of RLR signaling by targeting MAVS. Cell.

[52] Wolf A.J., Reyes C.N., Liang W., Becker C., Shimada K., Wheeler M.L., Cho H.C., Popescu N.I., Coggeshall K.M., Arditi M. (2016). Hexokinase is an innate immune receptor for the detection of bacterial peptidoglycan. Cell.

[53] Liberti M.V., Dai Z., Wardell S.E., Baccile J.A., Liu X., Gao X., Baldi R., Mehrmohamadi M., Johnson M.O., Madhukar N.S. (2017). A predictive model for selective targeting of the Warburg effect through GAPDH inhibition with a natural product. Cell Metab..

[54] Shestov A.A., Liu X., Ser Z., Cluntun A.A., Hung Y.P., Huang L., Kim D., Le A., Yellen G., Albeck J.G. (2014). Quantitative determinants of aerobic glycolysis identify flux through the enzyme GAPDH as a limiting step. eLife.

[55] Nagy E., Rigby W.F.C. (1995). Glyceraldehyde-3-phosphate dehydrogenase selectively binds AU-rich RNA in the NAD+-binding region (Rossmann fold). J. Biol. Chem..

[56] Chang C.H., Curtis J.D., Maggi L.B., Faubert B., Villarino A.V., O’Sullivan D., Huang S.C.C., van der Windt G.J.W., Blagih J., Qiu J. (2013). Posttranscriptional control of T cell effector function by aerobic glycolysis. Cell.

[57] Millet P., Vachharajani V., McPhail L., Yoza B., McCall C.E. (2016). GAPDH binding to TNF-alpha mRNA contributes to posttranscriptional repression in monocytes: A novel mechanism of communication between inflammation and metabolism. J. Immunol..

[58] Galván-Peña S., Carroll R.G., Newman C., Hinchy E.C., Palsson-McDermott E., Robinson E.K., Covarrubias S., Nadin A., James A.M., Haneklaus M. (2019). Malonylation of GAPDH is an inflammatory signal in macrophages. Nat. Commun..

[59] Yun J., Mullarky E., Lu C., Bosch K.N., Kavalier A., Rivera K., Roper J., Chio I.I.C., Giannopoulou E.G., Rago C. (2015). Vitamin C selectively kills KRAS and BRAF mutant colorectal cancer cells by targeting GAPDH. Science.

[60] Kornberg M.D., Bhargava P., Kim P.M., Putluri V., Snowman A.M., Putluri N., Calabresi P.A., Snyder S.H. (2018). Dimethyl fumarate targets GAPDH and aerobic glycolysis to modulate immunity. Science.

[61] Liao S.T., Han C., Xu D.Q., Fu X.W., Wang J.S., Kong L.Y. (2019). 4-Octyl itaconate inhibits aerobic glycolysis by targeting GAPDH to exert anti-inflammatory effects. Nat. Commun..

[62] Bollong M.J., Lee G., Coukos J.S., Yun H., Zambaldo C., Chang J.W., Chin E.N., Ahmad I., Chatterjee A.K., Lairson L.L. (2018). A metabolite-derived protein modification integrates glycolysis with KEAP1–NRF2 signalling. Nature.

[63] Eberhard T., Kronvall G., Ullberg M. (1999). Surface bound plasmin promotes migration of Streptococcus pneumoniae through reconstituted basement membranes. Microb. Pathog..

[64] Bergmann S., Wild D., Diekmann O., Frank R., Bracht D., Chhatwal G.S., Hammerschmidt S. (2003). Identification of a novel plasmin(ogen)-binding motif in surface displayed α-enolase of Streptococcus pneumoniae. Mol. Microbiol..

[65] Sha J., Erova T.E., Alyea R.A., Wang S., Olano J.P., Pancholi V., Chopra A.K. (2009). Surface-expressed enolase contributes to the pathogenesis of clinical isolate ssu of Aeromonas hydrophilaa. J. Bacteriol..

[66] Wygrecka M., Marsh L.M., Morty R.E., Henneke I., Guenther A., Lohmeyer J., Markart P., Preissner K.T. (2009). Enolase-1 promotes plasminogen-mediated recruitment of monocytes to the acutely inflamed lung. Blood.

[67] De Rosa V., Galgani M., Porcellini A., Colamatteo A., Santopaolo M., Zuchegna C., Matarese G. (2015). Glycolysis controls the induction of human regulatory T cells by modulating the expression of FOXP3 exon 2 splicing variants. Nat. Immunol..

[68] Tamada M., Suematsu M., Saya H. (2012). Pyruvate kinase M2: Multiple faces for conferring benefits on cancer cells. Clin. Cancer Res..

[69] Cao Y., Rathmell J.C., Macintyre A.N. (2014). Metabolic reprogramming towards aerobic glycolysis correlates with greater proliferative ability and resistance to metabolic inhibition in CD8 versus CD4 T cells. PLoS ONE.

[70] Palsson-Mcdermott E.M., Curtis A.M., Goel G., Lauterbach M.A.R., Sheedy F.J., Gleeson L.E., van den Bosch M.W.M., Quinn S.R., Domingo-Fernandez R., Johnson D.G.W. (2015). Pyruvate kinase M2 regulates hif-1α activity and il-1β induction and is a critical determinant of the Warburg effect in LPS-activated macrophages. Cell Metab..

[71] He C.L., Bian Y.Y., Xue Y., Liu Z.X., Zhou K.Q., Yao C.F., Lin Y., Zou H.F., Luo F.X., Qu Y.Y. (2016). Pyruvate Kinase M2 Activates mTORC1 by Phosphorylating AKT1S1. Sci. Rep..

[72] Ye J., Mancuso A., Tong X., Ward P.S., Fan J., Rabinowitz J.D., Thompson C.B. (2012). Pyruvate kinase M2 promotes de novo serine synthesis to sustain mTORC1 activity and cell proliferation. Proc. Natl. Acad. Sci. USA.

[73] Luo W., Hu H., Chang R., Zhong J., Knabel M., O’Meally R., Cole R.N., Pandey A., Semenza G.L. (2011). Pyruvate kinase M2 is a PHD3-stimulated coactivator for hypoxia-inducible factor 1. Cell.

[74] Angiari S., Runtsch M.C., Sutton C.E., Pearce E.L., Mills K.H.G., O’neill L.A.J. (2020). Pharmacological activation of pyruvate kinase M2 inhibits T Cell pathogenicity and suppresses autoimmunity. Cell Metab..

[75] Kono M., Maeda K., Stocton-Gavanescu I., Pan W., Umeda M., Katsuyama E., Burbano C., Orite S.Y.K., Vukelic M., Tsokos M.G. (2019). Pyruvate kinase M2 is requisite for Th1 and Th17 differentiation. JCI Insight.

[76] Damasceno L.E.A., Prado D.S., Veras F.P., Fonseca M.M., Toller-Kawahisa J.E., Rosa M.H., Alves-Filho J.C. (2020). PKM2 promotes Th17 cell differentiation and autoimmune inflammation by fine-tuning STAT3 activation. J. Exp. Med..

[77] Pucino V., Certo M., Bombardieri M., Pitzalis C. (2019). Lactate buildup at the site of chronic inflammation promotes disease by inducing CD4+ T Cell Metabolic Rewiring. Cell Metab..

[78] Xie M., Yu Y., Kang R., Zhu S., Yang L., Zeng L., Sun X., Yang M., Billiar T.R., Wang H. (2016). PKM2-Dependent glycolysis promotes NLRP3 and AIM2 inflammasome activation. Nat. Commun..

[79] Walls J.F., Subleski J.J., Palmieri E.M., Gonzalez Cotto M., Gardiner C.M., McVicar D.W., Finlay D.K. (2020). Metabolic but not transcriptional regulation by PKM2 is important for Natural Killer cell responses. eLife.

[80] Menk A., Scharping N.E., Moreci R.S., Zeng X., Guy C., Salvatore S., Bae H., Xie J., Young H.A., Wendell S.G. (2018). Early TCR signaling induces rapid aerobic glycolysis enabling distinct acute T cell effector functions. Cell Rep..

[81] Gerriets V.A., Kishton R.J., Nichols A.G., MacIntyre A.N., Inoue M., Ilkayeva O., Winter P.S., Liu X., Priyadharshini B., Slawinska M.E. (2015). Metabolic programming and PDHK1 control CD4+ T cell subsets and inflammation. J. Clin. Investig..

[82] Bian L., Josefsson E., Jonsson I.M., Verdrengh M., Ohlsson C., Bokarewa M., Tarkowski A., Magnusson M. (2009). Dichloroacetate alleviates development of collagen II-induced arthritis in female DBA/1 mice. Arthritis Res..

[83] Ostroukhova M., Goplen N., Karim M.Z., Michalec L., Guo L., Liang Q., Alam R. (2012). The role of low-level lactate production in airway inflammation in asthma. Am. J. Physiol. Lung Cell. Mol. Physiol..

[84] Peng M., Yin N., Chhangawala S., Xu K., Leslie C.S., Li M.O. (2016). Aerobic glycolysis promotes T helper 1 cell differentiation through an epigenetic mechanism. Science.

[85] Kaushik D.K., Bhattacharya A., Mirzaei R., Rawji K.S., Ahn Y., Rho J.M., Yong V.W. (2019). Enhanced glycolytic metabolism supports transmigration of brain-infiltrating macrophages in multiple sclerosis. J. Clin. Investig..

[86] Pioli P.A., Jonell Hamilton B., Connolly J.E., Brewer G., Rigby W.F.C. (2002). Lactate dehydrogenase is an AU-rich element-binding protein that directly interacts with AUF1. J. Biol. Chem..

[87] Calcinotto A., Filipazzi P., Grioni M., Iero M., De Milito A., Ricupito A., Rivoltini L. (2012). Modulation of microenvironment acidity reverses anergy in human and murine tumor-infiltrating T lymphocytes. Cancer Res..

[88] Fischer K., Hoffmann P., Voelkl S., Meidenbauer N., Ammer J., Edinger M., Kreutz M. (2007). Inhibitory effect of tumor cell-derived lactic acid on human T cells. Blood.

[89] Angelin A., Gil-de-Gómez L., Dahiya S., Jiao J., Guo L., Levine M.H., Beier U.H. (2017). Foxp3 Reprograms T Cell Metabolism to Function in Low-Glucose, High-Lactate Environments. Cell Metab..

[90] Comito G., Iscaro A., Bacci M., Morandi A., Ippolito L., Parri M., Chiarugi P. (2019). Lactate modulates CD4 + T-cell polarization and induces an immunosuppressive environment, which sustains prostate carcinoma progression via TLR8/miR21 axis. Oncogene.

[91] Colegio O.R., Chu N.Q., Szabo A.L., Chu T., Rhebergen A.M., Jairam V., Medzhitov R. (2014). Functional polarization of tumour-associated macrophages by tumour-derived lactic acid. Nature.

[92] Liu N., Luo J., Kuang D., Xu S., Duan Y., Xia Y., Yang X.P. (2019). Lactate inhibits ATP6V0d2 expression in tumor-associated macrophages to promote HIF-2α–mediated tumor progression. J. Clin. Investig..

[93] Bohn T., Rapp S., Luther N., Klein M., Bruehl T.J., Kojima N., Bopp T. (2018). Tumor immunoevasion via acidosis-dependent induction of regulatory tumor-associated macrophages. Nat. Immunol..

[94] Zhang D., Tang Z., Huang H., Zhou G., Cui C., Weng Y., Liu W., Kim S., Lee S., Perez-Neut M. (2019). Metabolic regulation of gene expression by histone lactylation. Nature.

[95] Mehta M.M., Weinberg S.E., Chandel N.S. (2017). Mitochondrial control of immunity: Beyond ATP. Nat. Rev. Immunol..

[96] Bailis W., Shyer J.A., Zhao J., Canaveras J.C.G., al Khazal F.J., Qu R., Steach H.R., Bielecki P., Khan O., Jackson R. (2019). Distinct modes of mitochondrial metabolism uncouple T cell differentiation and function. Nature.

[97] Ryan D.G., O’Neill L.A.J. (2020). Krebs cycle reborn in macrophage immunometabolism. Annu. Rev. Immunol..

[98] Yamada K.J., Heim C.E., Xi X., Attri K.S., Wang D., Zhang W., Singh P.K., Bronich T.K., Kielian T. (2020). Monocyte metabolic reprogramming promotes pro-inflammatory activity and Staphylococcus aureus biofilm clearance. PLoS Pathog..

[99] Bailey J.D., Diotallevi M., Nicol T., McNeill E., Shaw A., Chuaiphichai S., Hale A., Starr A., Nandi M., Stylianou E. (2019). Nitric Oxide Modulates Metabolic Remodeling in Inflammatory Macrophages through TCA Cycle Regulation and Itaconate Accumulation. Cell Rep..

[100] Palmieri E.M., Gonzalez-Cotto M., Baseler W.A., Davies L.C., Ghesquière B., Maio N., Rice C.M., Rouault T.A., Cassel T., Higashi R.M. (2020). Nitric oxide orchestrates metabolic rewiring in M1 macrophages by targeting aconitase 2 and pyruvate dehydrogenase. Nat. Commun..

[101] Infantino V., Convertini P., Cucci L., Panaro M.A., di Noia M.A., Calvello R., Palmieri F., Iacobazzi V. (2011). The mitochondrial citrate carrier: A new player in inflammation. Biochem. J..

[102] Wellen K.E., Hatzivassiliou G., Sachdeva U.M., Bui T.V., Cross J.R., Thompson C.B. (2009). ATP-citrate lyase links cellular metabolism to histone acetylation. Science.

[103] Meiser J., Krämer L., Sapcariu S.C., Battello N., Ghelfi J., D’Herouel A.F., Skupin A., Hiller K. (2016). Pro-inflammatory macrophages sustain pyruvate oxidation through pyruvate dehydrogenase for the synthesis of itaconate and to enable cytokine expression. J. Biol. Chem..

[104] Witkowski A., Thweatt J., Smith S. (2011). Mammalian ACSF3 protein is a malonyl-CoA synthetase that supplies the chain extender units for mitochondrial fatty acid synthesis. J. Biol. Chem..

[105] O’Neill L.A.J., Artyomov M.N. (2019). Itaconate: The poster child of metabolic reprogramming in macrophage function. Nat. Rev. Immunol..

[106] Tallam A., Perumal T.M., Antony P.M., Jäger C., Fritz J.V., Vallar L., Balling R., del Sol A., Michelucci A. (2016). Gene regulatory network inference of immunoresponsive gene 1 (IRG1) identifies interferon regulatory factor 1 (IRF1) as its transcriptional regulator in mammalian macrophages. PLoS ONE.

[107] Michelucci A., Cordes T., Ghelfi J., Pailot A., Reiling N., Goldmann O., Binz T., Wegner A., Tallam A., Rausell A. (2013). Immune-responsive gene 1 protein links metabolism to immunity by catalyzing itaconic acid production. Proc. Natl. Acad. Sci. USA.

[108] Li R., Zhang P., Wang Y., Tao K. (2020). Itaconate: A metabolite regulates inflammation response and oxidative stress. Oxidative Med. Cell. Longev..

[109] Lampropoulou V., Sergushichev A., Bambouskova M., Nair S., Vincent E.E., Loginicheva E., Cervantes-Barragan L., Ma X., Huang S.C.C., Griss T. (2016). Itaconate links inhibition of succinate dehydrogenase with macrophage metabolic remodeling and regulation of inflammation. Cell Metab..

[110] Mills E.L., Ryan D.G., Prag H.A., Dikovskaya D., Menon D. (2018). Itaconate is an anti-inflammatory metabolite that activates Nrf2 via alkylation of KEAP1. Nature.

[111] Mills E.L., Kelly B., Logan A., Costa A.S.H., Varma M., Bryant C.E., Tourlomousis P., Däbritz J.H.M., Gottlieb E., Latorre I. (2016). Succinate dehydrogenase supports metabolic repurposing of mitochondria to drive inflammatory macrophages. Cell.

[112] Tarasenko T.N., Pacheco S.E., Koenig M.K., Gomez-Rodriguez J., Kapnick S.M., Diaz F., Zerfas P.M., Barca E., Sudderth J., DeBerardinis R.J. (2017). Cytochrome c oxidase activity is a metabolic checkpoint that regulates cell fate decisions during T cell activation and differentiation. Cell Metab..

[113] Sena L.A., Li S., Jairaman A., Prakriya M., Ezponda T., Hildeman D.A., Wang C.R., Schumacker P.T., Licht J.D., Perlman H. (2013). Mitochondria are required for antigen-specific t cell activation through reactive oxygen species signaling. Immunity.

[114] Garaude J., Acín-Pérez R., Martínez-Cano S., Enamorado M., Ugolini M., Nistal-Villán E., Hervás-Stubbs S., Pelegrín P., Sander L.E., Enríquez J.A. (2016). Mitochondrial respiratory-chain adaptations in macrophages contribute to antibacterial host defense. Nat. Immunol..

[115] Clementi E., Brown G.C., Feelisch M., Moncada S. (1998). Persistent inhibition of cell respiration by nitric oxide: Crucial role of S-nitrosylation of mitochondrial complex I and protective action of glutathione. Proc. Natl. Acad. Sci. USA.

[116] NCT02762500. NCT02762500.

[117] Puleston D.J., Buck M.D., Klein R.I., Pearce E.J., Balabanov S., Pearce E.L. (2019). Polyamines and eIF5A hypusination modulate mitochondrial respiration and macrophage activation. Cell Metab..

[118] Park M.H., Wolff E.C. (2018). Hypusine, a polyamine-derived amino acid critical for eukaryotic translation. J. Biol. Chem..

[119] Zhao R.Z., Jiang S., Zhang L., Yu Z. (2019). Mitochondrial electron transport chain, ROS generation and uncoupling (Review). Int. J. Mol. Med..

[120] Cameron A.M., Castoldi A., Sanin D.E., Flachsmann L.J., Field C.S., Puleston D.J., Kyle R.L., Patterson A.E., Hässler F., Buescher J.M. (2019). Inflammatory macrophage dependence on NAD + salvage is a consequence of reactive oxygen species–mediated DNA damage. Nat. Immunol..

[121] Zhang D., Jin W., Wu R., Li J., Park S.A., Tu E., Zanvit P., Xu J., Liu O., Cain A. High Glucose Intake Exacerbates Autoimmunity through Reactive-Oxygen-Species-Mediated TGF-β Cytokine Activation. Immunity..

[122] Park M., Lee S., Moon S. (2016). Metformin attenuates graft-versus-host disease via restricting mammalian target of rapamycin/signal transducer and activator of transcription 3 and promoting adenosine monophosphate-activated protein kinase-autophagy for the balance between T helper 17 and Tregs. Transl. Res..

[123] O’Neill L.A.J., Kishton R.J., Rathmell J. (2016). A guide to immunometabolism for immunologists. Nat. Rev. Immunol..

[124] Nagy C., Haschemi A. (2015). Time and demand are two critical dimensions of immunometabolism: The process of macrophage activation and the pentose phosphate pathway. Front. Immunol..

[125] Gaber T., Strehl C., Buttgereit F. (2017). Metabolic regulation of inflammation. Nat. Rev. Rheumatol..

[126] Haschemi A., Kosma P., Gille L., Evans C.R., Burant C.F., Starkl P., Knapp B., Haas R., Schmid J.A., Jandl C. (2012). The sedoheptulose kinase CARKL directs macrophage polarization through control of glucose metabolism. Cell Metab..

[127] Oronsky B., Scicinski J., Ning S., Peehl D., Oronsky A., Cabrales P., Bednarski M., Knox S. (2016). RRx-001, A novel dinitroazetidine radiosensitizer. Investig. New Drugs.

[128] Michalek R.D., Gerriets V.A., Jacobs S.R., Macintyre A.N., MacIver N.J., Mason E.F., Sullivan S.A., Nichols A.G., Rathmell J.C. (2011). Cutting edge: Distinct glycolytic and lipid oxidative metabolic programs are essential for effector and regulatory CD4 + T Cell Subsets. J. Immunol..

[129] van der Windt G.J.W., Everts B., Chang C.H., Curtis J.D., Freitas T.C., Amiel E., Pearce E.J., Pearce E.L. (2012). Mitochondrial respiratory capacity is a critical regulator of cd8 + t cell memory development. Immunity.

[130] Raud B., Roy D.G., Divakaruni A.S. (2018). Etomoxir actions on regulatory and memory T cells are independent of Cpt1a-mediated fatty acid oxidation. Cell Metab..

[131] O’Sullivan D., vanderWindt G.W.J., Huang S.C.C., Curtis J.D., Chang C.H., Buck M.D.L., Qiu J., Smith A.M., Lam W.Y., DiPlato L.M. (2014). Memory CD8 + T Cells use cell-intrinsic lipolysis to support the metabolic programming necessary for development. Immunity.

[132] Malandrino M.I., Fucho R., Weber M., Calderon-Dominguez M., Mir J.F., Valcarcel L., Escoté X., Gómez-Serrano M., Peral B., Salvadó L. (2015). Enhanced fatty acid oxidation in adipocytes and macrophages reduces lipid-induced triglyceride accumulation and inflammation. Am. J. Physiol. Endocrinol. Metab..

[133] Shriver L.P., Manchester M. (2011). Inhibition of fatty acid metabolism ameliorates disease activity in an animal model of multiple sclerosis. Sci. Rep..

[134] Byersdorfer C.A., Tkachev V., Opipari A.W., Goodell S., Swanson J., Sandquist S., Glick G.D., Ferrara J.L.M. (2013). Effector T cells require fatty acid metabolism during murine graft-versus-host disease. Blood.

[135] Everts B., Amiel E., Huang S.C.C., Smith A.M., Chang C.H., Lam W.Y., Redmann V., Freitas T.C., Blagih J., van der Windt G.J.W. (2014). TLR-driven early glycolytic reprogramming via the kinases TBK1-IKKε supports the anabolic demands of dendritic cell activation. Nat. Immunol..

[136] Angela M., Endo Y., Asou H.K., Yamamoto T., Tumes D.J., Tokuyama H., Yokote K., Nakayama T. (2016). Fatty acid metabolic reprogramming via mTOR-mediated inductions of PPARγ directs early activation of T cells. Nat. Commun..

[137] Yang W., Bai Y., Xiong Y., Zhang J., Chen S., Zheng X., Meng X., Li L., Wang J., Xu C. (2016). Potentiating the antitumour response of CD8+ T cells by modulating cholesterol metabolism. Nature.

[138] Lee J., Walsh M.C., Hoehn K.L., James D.E., Wherry E.J., Choi Y. (2014). Regulator of fatty acid metabolism, acetyl coenzyme a carboxylase 1, controls T cell immunity. J. Immunol..

[139] Berod L., Friedrich C., Nandan A., Freitag J., Hagemann S., Harmrolfs K., Sandouk A., Hesse C., Castro C.N., BäHre H. (2014). De novo fatty acid synthesis controls the fate between regulatory T and T helper 17 cells. Nat. Med..

[140] Fox J.T., Stover P.J. (2008). Chapter 1 Folate-Mediated One-Carbon Metabolism. Vitam. Horm..

[141] Ducker G.S., Rabinowitz J.D. (2017). One-carbon metabolism in health and disease. Cell Metab..

[142] Ron-Harel N., Santos D., Ghergurovich J.M., Sage P.T., Reddy A., Lovitch S.B., Dephoure N., Satterstrom F.K., Sheffer M., Spinelli J.B. (2016). Mitochondrial biogenesis and proteome remodeling promote one-carbon metabolism for T cell activation. Cell Metab..

[143] Ma E.H., Bantug G., Griss T., Condotta S., Johnson R.M., Samborska B., Mainolfi N., Suri V., Guak H., Balmer M.L. (2017). Serine is an essential metabolite for effector t cell expansion. Cell Metab..

[144] Ron-Harel N., Notarangelo G., Ghergurovich J.M., Paulo J.A., Sage P.T., Santos D., Kyle Satterstrom F., Gygi S.P., Rabinowitz J.D., Sharpe A.H. (2018). Defective respiration and one-carbon metabolism contribute to impaired naïve T cell activation in aged mice. Proc. Natl. Acad. Sci. USA.

[145] Yu W., Wang Z., Zhang X., Wu Y., Correspondence D.W. (2019). One-carbon metabolism supports S-adenosylmethionine and histone methylation to drive inflammatory macrophages. Mol. Cell.

[146] Rodriguez A.E., Ducker G.S., Billingham L.K., Martinez C.A., Mainolfi N., Suri V., Friedman A., Manfredi M.G., Weinberg S.E., Rabinowitz J.D. (2019). Serine metabolism supports macrophage IL-1β production. Cell Metab..

[147] Ducker G.S., Chen L., Morscher R.J., Ghergurovich J.M., Esposito M., Teng X., Kang Y., Rabinowitz J.D. (2016). Reversal of cytosolic one-carbon flux compensates for loss of the mitochondrial folate pathway. Cell Metab..

[148] Pålsson-McDermott E.M., O’Neill L.A.J. (2020). Targeting immunometabolism as an anti-inflammatory strategy. Cell Res..

[149] Sinclair L.V., Rolf J., Emslie E., Shi Y.B., Taylor P.M., Cantrell D.A. (2013). Control of amino-acid transport by antigen receptors coordinates the metabolic reprogramming essential for T cell differentiation. Nat. Immunol..

[150] Nakaya M., Xiao Y., Zhou X., Chang J.H., Chang M., Cheng X., Blonska M., Lin X., Sun S.C. (2014). Inflammatory T cell responses rely on amino acid transporter ASCT2 facilitation of glutamine uptake and mTORC1 kinase activation. Immunity.

[151] Srivastava M.K., Sinha P., Clements V.K., Rodriguez P., Ostrand-Rosenberg S. (2010). Myeloid-derived suppressor cells inhibit T-cell activation by depleting cystine and cysteine. Cancer Res..

[152] Munn D.H., Zhou M., Attwood J.T., Bondarev I., Conway S.J., Marshall B., Brown C., Mellor A.L. (1998). Prevention of allogeneic fetal rejection by tryptophan catabolism. Science.

[153] Sedlmayr P., Blaschitz A., Stocker R. (2014). The role of placental tryptophan catabolism. Front. Immunol..

[154] Triplett T.A., Garrison K.C., Marshall N., Donkor M., Blazeck J., Lamb C., Qerqez A., Dekker J.D., Tanno Y., Lu W.C. (2018). Reversal of indoleamine 2,3-dioxygenase–Mediated cancer immune suppression by systemic kynurenine depletion with a therapeutic enzyme. Nat. Biotechnol..

[155] Minhas P.S., Liu L., Moon P.K., Joshi A.U., Dove C., Mhatre S., Contrepois K., Wang Q., Lee B.A., Coronado M. (2019). Macrophage de novo NAD+ synthesis specifies immune function in aging and inflammation. Nat. Immunol..

[156] Albina J.E., Mills C.D., Barbul A., Thirkill C.E., Henry W.L., Mastrofrancesco B., Caldwell M.D. (1988). Arginine metabolism in wounds. Am. J. Physiol. Endocrinol. Metab..

[157] El-Gayar S., Thüring-Nahler H., Pfeilschifter J., Röllinghoff M., Bogdan C. (2003). Translational control of inducible nitric oxide synthase by il-13 and arginine availability in inflammatory macrophages. J. Immunol..

[158] Duque-Correa M.A., Kühl A.A., Rodriguez P.C., Zedler U., Schommer-Leitner S., Rao M., Weiner J., Hurwitz R., Qualls J.E., Kosmiadi G.A. (2014). Macrophage arginase-1 controls bacterial growth and pathology in hypoxic tuberculosis granulomas. Proc. Natl. Acad. Sci. USA.

[159] Vigeland C.L., Beggs H.S., Collins S.L., Chan-Li Y., Powell J.D., Doerschuk C.M., Horton M.R. (2019). Inhibition of glutamine metabolism accelerates resolution of acute lung injury. Physiol. Rep..

[160] Leone R.D., Zhao L., Englert J.M., Sun I.M., Oh M.H., Sun I.H., Arwood M.L., Bettencourt I.A., Patel C.H., Wen J. (2019). Glutamine blockade induces divergent metabolic programs to overcome tumor immune evasion. Science.

[161] Altman B.J., Jacobs S.R., Mason E.F., Michalek R.D., MacIntyre A.N., Coloff J.L., Ilkayeva O., Jia W., He Y.W., Rathmell J.C. (2011). Autophagy is essential to suppress cell stress and to allow BCR-Abl-mediated leukemogenesis. Oncogene.

[162] Mcleod I.X., Jia W., He Y.W. (2012). The contribution of autophagy to lymphocyte survival and homeostasis. Immunol. Rev..

[163] Espert L., Denizot M., Grimaldi M., Robert-Hebmann V., Gay B., Varbanov M., Codogno P., Biard-Piechaczyk M. (2006). Autophagy is involved in T cell death after binding of HIV-1 envelope proteins to CXCR4. J. Clin. Investig..

[164] Riffelmacher T., Richter F.C., Simon A.K. (2018). Autophagy dictates metabolism and differentiation of inflammatory immune cells. Autophagy.

[165] Kang Y.H., Cho M.H., Kim J.Y., Kwon M.S., Peak J.J., Kang S.W., Yoon S.Y., Song Y. (2016). Impaired macrophage autophagy induces systemic insulin resistance in obesity. Oncotarget.

[166] Mouttie L.E., Vu T., Lineburg K.E., Kuns R.D., Bagger F.O., Teal B.E., Lor M., Boyle G.M., Bruedigam C., Mintern J.D. (2015). Autophagy is required for stem cell mobilization by G-CSF. Blood.

